# Open aortic arch reconstruction for acute type a aortic dissection: a single-center experience with 267 consecutive patients

**DOI:** 10.1186/s13019-016-0500-5

**Published:** 2016-07-22

**Authors:** Shuyang Lu, Shouguo Yang, Hao Lai, Jiayu Zheng, Tao Hong, Xiaoning Sun, Chunsheng Wang

**Affiliations:** Shanghai Institute of Cardiovascular Disease, Zhongshan Hospital, Fudan University, Shanghai, China; Department of Cardiovascular Surgery, Zhongshan Hospital, Fudan University, Shanghai, China; Fenglin Road 180, Xuhui District, Shanghai, 200032 China

**Keywords:** Type A aortic dissection, Open arch reconstruction, Mortality, Morbidity

## Abstract

**Background:**

This study aimed to analyze the mortality and morbidity of patients undergoing open aortic arch reconstruction for acute type A aortic dissection.

**Methods:**

Between September 2005 and January 2012, 267 consecutive patients underwent open aortic arch reconstruction for acute type A aortic dissection at our center. The mean age was 51.2 ± 10.0 years, and 200 patients were male. Sixty-three and 184 patients underwent hemiarch replacement and total arch replacement, respectively, whereas the remaining 20 patients underwent single- or triple-branched stent graft implantation. Long-term mortality was estimated by Kaplan–Meier method.

**Results:**

The in-hospital and operative mortality rates within 30 days were 11.2 % and 8.2 %, respectively. The cardiopulmonary bypass, myocardial ischemic, and antegrade cerebral perfusion times were 150.2 ± 43.3, 71.9 ± 33.2, and 33.6 ± 14.4 min, respectively. The overall in-hospital and intensive care unit durations and mean ventilation time were 23.9 ± 18.4 and 9.5 ± 12.7 days and 122.7 ± 183.4 h, respectively. We observed new postoperative permanent neurological dysfunction in 29 patients and temporary neurological dysfunction in 17 patients. The mean follow-up duration was 52.4 ± 27.9 months; 76.4 % of patients completed follow-up and 143 remained alive. Overall long-term survival was 82.2 % at 5 years.

**Conclusions:**

The open aortic arch reconstruction technique for acute type A dissection carries a relatively high in-hospital mortality risk, although the late results are encouraging. Patients with an advanced age or impaired renal function may opt for endovascular or modified single- or triple-branched stent graft implantation therapy.

## Background

Acute Type A aortic dissection involving the aortic arch remains a catastrophic condition for patients, with high mortality and morbidity rates [1, 2]. The International Registry of Acute Aortic Dissection data report in-hospital mortality rates as high as 26 % [3, 4]. Therefore, surgical treatment is usually required, and several surgical methods have been introduced. In recent years, although advancements in thoracic endovascular aortic repair such as branched endografts or hybrid-debranching endovascular aortic repair have extended the option of endoluminal therapy into the realm of the aortic arch, open aortic arch reconstruction currently remains an irreplaceable surgical technique. However, this technique remains a surgical challenge because of the long operation time, bleeding complications, and high morbidity and mortality resulting from multiple organ failure.

In the present study, we retrospectively investigated our experiences and reported the surgical mortality and morbidity rates and follow-up results of patients who underwent open aortic arch reconstruction for acute type A aortic dissection.

## Methods

The study protocol was approved by the Committee for the Protection of Human Subjects at the Zhongshan Hospital Fudan University (Shanghai, China). Informed consent was obtained from each patient involved in this study. The study design was a retrospective observational study.

### Patient demographics and characteristics

Between September 2005 and January 2012, 267 consecutive patients who underwent open aortic arch reconstruction for acute type A aortic dissection with hypothermic circulatory arrest either with or without antegrade cerebral perfusion (ACP) at Zhongshan Hospital (Shanghai, China) were included in the present study. The mean patient age was 51.2 ± 10.0 years (range, 26–75 years), and 200 patients were male. Concurrently, 22 patients with chronic type A aortic dissection also underwent open aortic arch reconstruction. Preoperative diagnoses were based on the computed tomography angiography and echocardiography results. Compared to patients with chronic type A aortic dissection, preoperative hypertension and diabetes mellitus were more common in patients with acute type aortic dissection (50 % versus 71.9 %, *p* = 0.05 and 4.5 % versus 24.3 %, *p* = 0.03, respectively). There were no significant differences in other preoperative medical disorders between the two groups. Additional details of the demographic and preoperative data are shown in Table 1.

**Table 1:** Preoperative characteristics

Characteristics	Acute type A dissection	Chronic type A dissection	*p* value
Number of patients	267	22	
Age (years)	51.2 ± 10.0 (26–75)	48.0 ± 9.7 (31–64)	0.17
Gender			
Male	200 (74.9 %)	16 (72.7 %)	1.00
Female	67 (25.1 %)	6 (27.3 %)	1.00
Hypertension	192 (71.9 %)	11 (50 %)	0.05
DM	65 (24.3 %)	1 (4.5 %)	0.03
Previous CVA	1 (0.4 %)	0	1.00
PVD	3 (1.1 %)	0	1.00
Renal dysfunction	29 (7.9 %)	1 (4.5 %)	0.71
COPD	3 (1.1 %)	0	1.00
Previous cardiac surgery	6 (2.2 %)	2 (9.0 %)	0.11
Previous EVR	12 (4.5 %)	0	0.61
Marfan syndrome	8 (3.0 %)	1 (4.5 %)	0.52
BAV	8 (3.0 %)	0	1.00

Continuous variables are reported as means ± standard deviations; categorical variables are reported as numbers and percentages

Serum creatinine >2.0 mg/mL

*DM* diabetes mellitus, *CVA* cerebrovascular accident, *PVD* peripheral vascular disease, *COPD* chronic obstructive pulmonary disease, *EVR* endovascular repair, *BAV* bicuspid aortic valve

The indications for open aortic arch reconstruction were any of the following conditions: (1) a primary tear located in the transverse arch or proximal descending aorta; (2) cerebral system malperfusion symptoms; or (3) the presence of Marfan syndrome. The following patients were excluded from this study: those in whom only the partial ascending aorta was replaced without arch intervention and those with severe preoperative neurological dysfunction.

### Operative technique

Our approach to open aortic arch reconstruction was described previously in detail [5, 6]. All patients received general anesthesia via the intravenous administration of narcotics and muscle relaxants. The left radial artery and dorsal artery of the foot were cannulated for continuous blood pressure monitoring. Temperature probes were placed for esophagopharyngeal and bladder temperature monitoring. The surgical technique included a total cardiopulmonary bypass (CPB), profound hypothermia, and circulatory arrest with or without ACP. Circulatory arrest was initiated when the nasopharyngeal temperature reached 16–20 °C, and pharmacologic agents (thiamylal sodium, phenytoin, and mannitol) were administered for brain protection in addition to placing ice packs around the head. The right axillary artery was dissected for arterial perfusion and unilateral ACP. The right femoral artery was also occasionally dissected for arterial perfusion. Bilateral ACP was usually performed through the right axillary artery and the left common carotid artery. The flow rate was maintained at 8–10 ml/kg/min, and the perfusion pressure was maintained at 40–50 mmHg. The left subclavian artery was usually cross-clamped during ACP to achieve a bloodless surgical field. The arch reconstruction strategy was mainly based on an intraoperative inspection, including the location of the primary tear, extent of the tear, texture of the vascular wall, and situation of the supra-arch vessels. Briefly, a stent graft (MicroPort Medical Co Ltd, Shanghai, China) and a 4-branch or straight prosthetic graft (Boston Scientific Inc, Boston, MA) were used in total arch replacement combined with SET implantation. Proximal aortic repair was completed during the systematic rewarming period and was based on a surgical inspection of the aortic root involvement, including the aortic valve leaflets and the coronary ostia.

The single- and triple-branched stent graft implantation technique has been reported previously [7, 8]. The single-branched stent grafts were handmade and constructed by adding a sidearm stent graft to a conventional straight aortic stent graft. The conventional straight aortic stent graft (Microport Medical Corp, Shanghai, China) was 100 mm long and 26 to 30 mm in diameter. The sidearm stent graft (Yuhengjia Science and Technology Co Ltd, Beijing, China) was 25 mm long and 10 to 14 mm in diameter. The triple-branched stent graft was a branched 1-piece graft consisting of a self-expandable nitinol stent and polyester vascular graft fabric (Yuhengjia Sci Tech Corp Ltd, Beijing, China). This apparatus comprised a main graft and three sidearm grafts. The tapered main graft was 145 mm in length, 30 mm in proximal diameter, and 26 mm in distal diameter. A 10-mm-long stent-free sewing Dacron tube was present at the proximal end. The first sidearm graft was 35 mm long and 14 or 16 mm in diameter. Both the second and third sidearm grafts were 25 mm long and 12 or 14 mm in diameter. The distance between the two neighboring sidearm grafts was 3 mm. The main graft and three sidearm grafts were individually mounted on four catheters and restrained by four silk strings. All procedures were performed with patients under general anesthesia. The arterial blood pressure of both the upper and lower limbs was monitored, and a probe for transesophageal echocardiographic monitoring was inserted. A median sternotomy was performed in all patients. cardiopulmonary bypass was established by two venous cannulas through the right atrium and two arterial return cannulas placed in the femoral and right axillary arteries. A left ventricular vent was inserted through the right superior pulmonary vein. Systemic cooling was initiated while the ascending aorta, aortic arch vessels, and descending thoracic aorta were exposed. The ascending aorta was clamped at the base of the innominate artery and transected just above the sinotubular junction. Cardioplegia was infused directly into the both coronary arteries. Systemic cooling progressed until the patient’s rectal temperature reached below 25 °C, and the pharmacologic agents (thiamylal sodium, phenytoin, and mannitol) were administered for brain protection in addition to ice packs placed around the head, before circulatory arrest. After circulatory arrest had been instituted, the ascending aorta was unclamped and the aortic arch was opened. The main graft of the triple-branched stent graft was inserted into the true lumen of the arch and proximal descending aorta, and each sidearm was subsequently positioned individually into the left subclavian, left carotid, and brachio-cephalic arteries, respectively. After placing the main graft and sidearm grafts accurately, the restraining strings were withdrawn and the grafts were deployed. After the release of the aortic arch stent, inserted into 60° saline gauze in the lumen, the shape memory alloy stent fully open adherent, and 3-0 Prolene line with mat near the innominate artery, left subclavian artery and the main vessel stent through the suture arterial wall to prevent the late shift and poor adhesion. The transected distal stump of the ascending aorta was reconstructed by the inner proximal stent-free Dacron tube of the main graft, and subsequently continuous anastomosis to the Dacron tube graft was made in an end-to-end fashion. Finally, the air was carefully flushed out from the triple-branched stent graft with femoral and right axillary blood return. Then, antegrade systemic perfusion from the branch of the Dacron tube graft was started, and the patient was rewarmed.

### Definitions

The following definitions were used in our study. Patients were considered to be in the acute phase of aortic dissection if therapy was performed within 2 weeks of symptom onset, in the subacute phase if therapy was instituted from 2 weeks to 2 months after symptom onset, and in the chronic phase if the symptoms had persisted for longer than 2 months. Temporary neurologic dysfunction (TND) was defined as the presence of a reversible postoperative motor deficit, confusion, agitation, or transient delirium. Permanent neurologic dysfunction (PND) was defined as the presence of either focal (stroke) or global (coma) permanent neurological deficits that persisted upon discharge from the hospital. Operative mortality was defined as death within 30 days after the operation. Overall hospital mortality was any death before hospital discharge and included patients who succumbed to operative death as defined above.

### Statistical analysis and follow-up

The surgical treatment technique allocation was not randomized but instead was determined according to the best medical judgment for each individual case. Data were collected from chart reviews and entered into a dedicated table in Microsoft Excel (Microsoft Corporation, Redmond, WA, USA). Continuous variables were presented as means ± standard deviations (SD). Comparisons between two groups were made with Student’s *t*-test, the Pearson *χ*^2^ test, or a nonparametric equivalent Mann–Whitney test. The significance level was set at *p* <0.05. The statistical analysis was performed using SPSS 14.0 software (SPSS Inc., Chicago, IL, USA).

## Results

### Operative data

Total arch replacement was performed in 184 acute type A aortic dissection patients and was more frequently performed in this group than in the chronic group (68.9 % versus 36.4 %, *p* <0.05). Subtotal arch replacement was performed in 63 patients in the acute group (23.6 %) and was less frequently performed in this group than in the chronic group (23.6 % versus 54.5 %, *p* <0.05). Twenty patients underwent open single- or triple-branched stent graft placement (7.7 %). The ascending aorta was replaced in all patients. Concomitant surgical procedures also included Bentall in 42 patients, David in nine patients, Wheat in seven patients, aortic valve plasty in six patients, and coronary artery bypass grafting in 25 patients. The detailed surgical strategies are summarized in Table 2.

**Table 2:** Surgical strategies

	Acute type A dissection	Chronic type A dissection	*p* value
Extent of aortic procedure			
AAR + HAR	63 (23.6 %)	12 (54.5 %)	0.00
AAR + TAR	184 (68.9 %)	8 (36.4 %)	0.00
AAR + single-branched stent graft implantation	6 (2.2 %)	2 (9.1 %)	0.12
AAR + triple-branched stent graft implantation	14 (5.2 %)	0	0.61
Elephant trunk technique	206 (77.2 %)	13 (59.1 %)	0.07
Concomitant procedures			
Bentall operation	42 (15.7 %)	8 (36.4 %)	0.03
David operation	9 (2.5 %)	2 (9.1 %)	0.20
Wheat operation	7 (1.9 %)	2 (9.1 %)	0.14
AVP	6 (2.2 %)	0	1.00
CABG	25 (9.4 %)	1 (4.5 %)	0.71

*AAR* ascending aorta replacement, *HAR* hemiarch replacement, *TAR* total arch replacement, *AVP* aortic valve plasty, *CABG* coronary artery bypass grafting

The total CPB times were 150.2 ± 43.3 and 182.4 ± 81.8 min for the acute and chronic type A dissection groups, respectively (*p* <0.05). The myocardial ischemic and ACP times in the acute and chronic groups were 71.9 ± 33.2 versus 80.1 ± 37.5 min and 33.6 ± 14.4 versus 34.5 ± 15.1 min, respectively (acute group versus chronic group, *p* = 0.31 and *p* = 0.80, respectively; Table 3). Before January 2009, we selected unilateral ACP combined with hypothermic circulatory arrest for cerebral protection. Bilateral ACP combined with hypothermic circulatory arrest was widely accepted after January 2009. Our recent study^5^ confirmed the safety of unilateral ACP with hypothermic circulatory arrest as a cerebral protection technique for open aortic arch reconstruction and determined that this technique was not inferior to bilateral ACP with hypothermic circulatory arrest. Therefore, we reconsidered the application of unilateral ACP combined with hypothermic circulatory arrest. For triple-branched stent graft placement technique, we usually used hypothermic circulatory arrest without cerebral perfusion because the entire procedure could be completed within 3–6 min [8].

**Table 3:** Perioperative data

	Acute type A dissection	Chronic type A dissection	*p* value
CPB time (minutes)	150.2 ± 43.3 (71–313)	182.4 ± 81.8 (95–484)	0.00
Cross-clamp time (minutes)	71.9 ± 33.2 (14–184)	80.1 ± 37.5 (78–106)	0.31
Cerebral perfusion time (minutes)	33.6 ± 14.4 (7–105)	34.5 ± 15.1 (11–64)	0.80
Ventilation time (hours)	122.7 ± 183.4 (6–1032)	51.5 ± 63.0 (11–264)	0.00
ICU time (days)	9.5 ± 12.7 (1–120)	5.1 ± 3.8 (2–15)	0.00
In-hospital time (days)	23.9 ± 18.4 (1–136)	20.9 ± 9.7 (7–44)	0.49
Nasopharyngeal temperature (°C)	16.8 ± 2.2 (10–24)	17.1 ± 2.2 (12–20.1)	0.63
Rectal temperature (°C)	21.6 ± 2.4 (13.4–27)	20.7 ± 2.2 (17.2–25.3)	0.16
RBC (ml)	1689.4 ± 1126.8 (300–7500)	1919.4 ± 1174.0 (450–5550)	0.41
Serum (ml)	1494.8 ± 1061.1 (0–9000)	1922.2 ± 1544.8 (200–7400)	0.11
Platelets (patients, %)	119 (44.6 %)	15 (68.2 %)	0.04
Platelets (packs)	0.5 ± 0.7 (0–4)	0.8 ± 0.7 (0–3)	0.05
First day drainage (ml)	510.1 ± 421.1 (60–2760)	749.4 ± 615.9 (200–2660)	0.03

*CPB* cardiopulmonary bypass, *ICU* intensive care unit, *RBC* red blood cells

The overall in-hospital durations of the acute and chronic groups were comparable (23.9 ± 18.4 versus 20.9 ± 9.7 days, *p* = 0.49), but the intensive care unit duration and mean ventilation time were much longer in the former group (9.5 ± 12.7 versus 5.1 ± 3.8 days, *p* <0.05 and 122.7 ± 183.4 versus 51.5 ± 63.0 h, *p* <0.05, respectively). The mean blood product usage volumes were 1689.4 ± 1126.8 ml (range, 300–7500 ml) for packed red blood cells and 1494.8 ± 1061.1 ml (range, 0–9000 ml) for serum. Additionally, 119 patients (44.6 %) required the transfusion of an average of 0.5 ± 0.7 platelet packs (range, 0–4 packs). The chest tube drainage volume within the first 24 postoperative hours was 510.1 ± 421.1 ml (Table 3).

### In-hospital mortality and morbidity

The operative mortality within 30 days was 8.2 %, and the hospital mortality was 11.2 % (Table 4). The causes of in-hospital death were multiple organ failure in 24 patients (including respiratory failure, renal failure, hepatic failure, stroke, and infection), low cardiac output syndrome in 1, sepsis in 3, massive gastrointestinal bleeding in 1, and secondary dissection rupture of the descending aorta in one patient.

**Table 4:** In-hospital mortality and morbidity

	Acute type A dissection	Chronic type A dissection	*p* value
In-hospital mortality	30 (11.2 %)	2 (9.0 %)	1.00
Operative mortality within 30 days	22 (8.2 %)	2 (9.0 %)	0.69
Renal failure	53 (19.8 %)	1 (4.5 %)	0.09
Required dialysis	49 (18.3 %)	1 (4.5 %)	0.14
Respiratory failure	78 (29.2 %)	3 (13.6 %)	0.14
Required tracheotomy	63 (23.6 %)	1 (4.5 %)	0.06
Re-operation for bleeding	4 (1.5 %)	1 (4.5 %)	0.33
Mediastinal infection	10 (3.7 %)	0	1.00
Paraplegia	5 (1.9 %)	0	1.00
TND	17 (6.4 %)	0	0.63
PND	29 (10.9 %)	2 (9.0 %)	1.00

*TND* temporary neurological dysfunction, *PND* permanent neurological dysfunction

Regarding new postoperative neurologic complications, PND occurred in 29 patients (10.9 %) and TND in 17 patients (6.4 %). The respiratory failure and renal dysfunction morbidities were 29.2 % (78/267) and 19.8 % (53/267), respectively. Tracheotomy or dialysis was required in 63 and 49 patients with respiratory failure and renal dysfunction, respectively. Most patients recovered and were successfully weaned from the ventilator after prolonged ventilation periods. Five patients (1.9 %) exhibited paraplegia until discharge. Four patients (1.5 %) required additional surgery to treat bleeding. Mediastinal infection occurred in 10 patients (3.7 %), three of who died as a result of sepsis; the others recovered after receiving anti-infection therapy. There were no significant differences between the acute and chronic groups. Details regarding morbidity are shown in Table 4.

### Follow-up

**Fig. 1:**
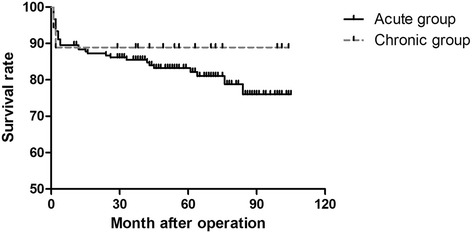
The overall long-term survival in patients with acute type A dissectoin compared with patients with chronic type A dissection at 5 years was 82.2 % versus 88.9 %

The overall long-term survival in patients with acute type A dissection compared with patients with chronic type A dissection at 5 years was 82.2 % versus 88.9 % (Fig. 1). The mean follow-up duration was 52.4 ± 27.9 months; 76.4 % of the patients completed follow-up and 143 remained alive. The major causes of late death included cardiac disease in five patients, malignancy in seven, cerebrovascular disease in five, secondary abdominal aortic dissection in four, renal failure in six, and unknown causes in 11 patients.

## Discussion

At present, the existence of considerable remaining debates and controversies regarding the treatment of acute type A aortic dissection is undeniable. In our present study, we reported our experience with a large sample of 267 cases of open aortic arch reconstruction for acute type A aortic dissection and found the results to be comparable with those in cases of chronic type A aortic dissection.

The elephant trunk technique is widely accepted as an effective treatment for type A dissection. To date, there have been many modifications and developments to the original elephant trunk technique. In an attempt to strengthen the distal anastomosis and facilitate secondary surgery on the distal aorta, Borst et al. [9] attempted to place the prosthetic graft into the true lumen of a type A aortic dissection, which was recognized as a conventional elephant trunk. Obviously, there are some limitations associated the conventional elephant trunk technique. First, it is very difficult to place a soft prosthetic graft into the small true lumen, and soft prosthetic grafts are prone to “flapping”; second, some patients die of secondary aortic dissection rupture before returning to the hospital for a second-stage procedure. Kato et al. [10] and Karck et al. [11] realized the shortcomings of the soft prosthetic graft and replaced it with a self-expandable frozen stent; however, this replacement was limited because only the distal part could be sustained by the stent. Recently, the reduced invasiveness of endovascular stent graft placement has made this an effective and popular option for the treatment of type B aortic dissection and for some selected patients, endoluminal therapy has extended even to the realm of type A dissection. In consideration of the advantages of endoluminal therapy, Sun et al. [1] modified the conventional elephant trunk technique to develop a long, self-expanded frozen stent that combined the advantages of both open surgical treatment and endoluminal therapy and reported excellent surgical and long-term follow-up results. However, even the modified long frozen stent developed by Sun and colleagues could not avoid the long period of circulatory arrest during total arch replacement. Therefore, Chen et al. [8] invented the triple-branched stent graft to simplify open aortic arch reconstruction for type A aortic dissection, thus allowing completion of the entire procedure within 3–6 min, with encouraging early results. In our present group, 206 patients (77.2 %) underwent the elephant trunk technique, including 186 who underwent Sun’s elephant trunk technique and 20 who underwent single- or triple-branched stent graft implantation. We agree with Chen et al. and hold the opinion that surgical technique development will tend toward simple, safe, and less invasive methods.

Cerebral protection is another issue that has remained controversial for many years. We have experience with hypothermic circulatory arrest, unilateral ACP, and bilateral ACP techniques and previously testified that unilateral ACP with hypothermic circulatory arrest is not inferior to bilateral ACP with hypothermic circulatory arrest. Currently, we tend to use unilateral ACP; however, we believe that perfusion pressure, flow rate, and cerebral oxygen saturation monitoring are indispensable for the real-time intraoperative monitoring of cerebral perfusion. If these measurements indicate insufficient cerebral perfusion, we can promptly add left carotid artery perfusion as needed. In our group, four cases were transferred to bilateral ACP during surgery because the intra-operative cerebral oxygen saturation was <50 %. However, the oxygen saturation levels in two cases remained below 50 % even with bilateral ACP, and these patients succumbed to postoperative coma. Kruger et al. [4] recognized that for cerebral protection, hypothermic circulatory arrest alone might suffice for circulatory arrest times <30 min and yield similar results as those achieved with ACP whereas for longer arrest times, the outcomes with unilateral and bilateral ACP were equivalent. Additionally, ACP is much popular than retrograde cerebral perfusion because it preserves the natural flow direction, allows higher pressures, and provides metabolically adequate perfusion. However, Perreas et al. [12] recently reported excellent results with the combined use of deep hypothermic circulatory arrest and retrograde cerebral perfusion and noted that this method was safe for ascending aorta and proximal aortic arch replacement. However, the mean total circulatory arrest time in that study was only 25.4 ± 13 min, which was not sufficient to highlight the importance of retrograde cerebral perfusion. We generally agree that hypothermic circulatory arrest alone might be sufficient for arrest times <30 min, but the combined use of this technique with other perfusion methods would increase the cerebral protection and safety. Furthermore, some authors have suggested a single-cannulation bilateral cerebral perfusion technique for type A dissection via the placement of a side-biting clamp behind the two supra-aortic arteries that allows the origins to freely communicate [13]. However, we think that this technique is improper for type A dissection for several reasons, which we have described previously [14].

Our results regarding in-hospital mortality and morbidity associated with acute type A aortic dissection arch reconstruction were comparable with those reported by other centers. Additionally, there were no significant differences between cases of acute and chronic type A aortic dissection as shown in Table 4. However, the present chronic A dissection group was much smaller than the acute A dissection group. On the other hand, the results of arch reconstruction for acute type A dissection have greatly improved in recent years along with technique development. For this reason, we observed lower levels of blood product usage and initial 24-h postoperative drainage in the acute A dissection group relative to the chronic group.

### Limitations

The present study has some inevitable limitations. First, this was a retrospective observational study, and the nonrandomized design might have affected the results through unrecognized confounding factors and biases. Second, this study represents only a single-center experience and not a multi-center experience. Disparities among different centers and operators certainly exist. Finally, the advances in surgical, anesthetic, cerebral protection, and perioperative care techniques have surely affected the surgical results associated with different time periods.

## Conclusions

The open aortic arch reconstruction technique for type A dissection carries a relatively high in-hospital mortality risk, although the late surgical results are encouraging. Along with the development of endoluminal therapy techniques such as single- or triple-branched stent graft implantation, patients with an advanced age or impaired renal function might become the most suitable candidates.

## Abbreviations

AAR: ascending aorta replacement
ACP: antegrade cerebral perfusion
AVP: aortic valve plasty
BAV: bicuspid aortic valve
COPD: chronic obstructive pulmonary disease
CPB: cardiopulmonary bypass
CVA: cerebrovascular accident
DM: diabetes mellitus
EVR: endovascular repair
HAR: hemi-arch replacement
ICU: intensive care unit
PND: permanent neurologic dysfunction
PVD: peripheral vascular disease
RBC: red blood cell
TAR: total arch replacement
TND: temporary neurologic dysfunction
